# Mammary tumors that become independent of the type I insulin-like growth factor receptor express elevated levels of platelet-derived growth factor receptors

**DOI:** 10.1186/1471-2407-11-480

**Published:** 2011-11-09

**Authors:** Craig I Campbell, Roger A Moorehead

**Affiliations:** 1 Department of Biomedical Sciences, University of Guelph, 50 Stone Rd. E, N1G2W1 Guelph, ON, Canada

**Keywords:** IGF-IR, PDGFR, Breast cancer, Migration, Resistance

## Abstract

**Background:**

Targeted therapies are becoming an essential part of breast cancer treatment and agents targeting the type I insulin-like growth factor receptor (IGF-IR) are currently being investigated in clinical trials. One of the limitations of targeted therapies is the development of resistant variants and these variants typically present with unique gene expression patterns and characteristics compared to the original tumor.

**Results:**

MTB-IGFIR transgenic mice, with inducible overexpression of the IGF-IR were used to model mammary tumors that develop resistance to IGF-IR targeting agents. IGF-IR independent mammary tumors, previously shown to possess characteristics associated with EMT, were found to express elevated levels of PDGFRα and PDGFRβ. Furthermore, these receptors were shown to be inversely expressed with the IGF-IR in this model. Using cell lines derived from IGF-IR-independent mammary tumors (from MTB-IGFIR mice), it was demonstrated that PDGFRα and to a lesser extent PDGFRβ was important for cell migration and invasion as RNAi knockdown of PDGFRα alone or PDGFRα and PDGFRβ in combination, significantly decreased tumor cell migration in Boyden chamber assays and suppressed cell migration in scratch wound assays. Somewhat surprisingly, concomitant knockdown of PDGFRα and PDGFRβ resulted in a modest increase in cell proliferation and a decrease in apoptosis.

**Conclusion:**

During IGF-IR independence, PDGFRs are upregulated and function to enhance tumor cell motility. These results demonstrate a novel interaction between the IGF-IR and PDGFRs and highlight an important, therapeutically relevant pathway, for tumor cell migration and invasion.

## Background

Targeting receptor tyrosine kinases (RTKs) for the treatment of breast cancer has emerged as a promising direction for the management of this disease. However, a major drawback of this line of treatment is the propensity of tumors to acquire resistance. For example, a number of mechanisms have been identified as mediators of resistance to the commonly used Her2-directed therapeutic trastuzumab (reviewed in [1]). Therefore, a powerful tool for overcoming this shortcoming and optimizing the use of such compounds will be understanding mechanisms of resistance.

The insulin-like growth factor (IGF)-axis comprises one such RTK signalling pathway heavily implicated in the progression of breast cancer and as such holds promise for therapeutic targeting. Upon activation by ligand (IGF-I or IGF-II), the IGF-IR undergoes tyrosine phosphorylation, subsequently signaling through a number of downstream pathways including MAPK and PI-3K/Akt. The mitogenicity of this receptor is well established, and is well known to contribute to transformation of normal mammary epithelial cells [2]. Effective targeting of this pathway was established in a number of xenograft models of breast cancer [3-7]. Activation of the IGF-IR is not correlated with any particular subtype of breast cancer [8] and therefore offers the opportunity for widespread use of targeted therapies. Based on these observations, a number of drugs targeting this pathway are currently in clinical trials [9,10], including small molecule kinase inhibitors that block downstream signalling and monoclonal antibodies that block ligand receptor interactions [11]. While it is too early to determine the efficacy of such agents [9], it is of extreme importance to understand potential resistance mechanisms.

Previously our lab has created an inducible transgenic mouse model (MTB-IGFIR) of IGF-IR overexpression in which mammary tumors were observed to develop [12]. IGF-IR transgene downregulation resulted in regression of almost all established palpable tumors after which a fraction recur in the absence of IGF-IR induction [13]. Thus, this model recapitulates treatment with IGF-IR-directed therapeutics and can be used to study potential resistance mechanisms. Previous work has provided initial characterization of IGF-IR-independence and has demonstrated the presence of an epithelial-mesenchymal transition (EMT) phenotype in these recurrent tumors [13]. Subsequent microarray data, comparing primary mammary tumors to IGF-IR-independent recurrent tumors, has since identified members of the platelet-derived growth factor family (PDGF) as being upregulated during this process [14].

The importance of PDGF receptors (PDGFRs) is well known in gliomas, sarcomas and gastrointestinal stromal tumors but has more recently been noted in such cancers as ovarian, prostate and neuroblastomas (reviewed in [15,16]). However, compared to other RTKs, little is known about the role of these proteins in breast cancer. Signalling through PDGFRs has been typically associated with proliferation and migration of mesenchymal cells and is important for these processes during mammalian development (reviewed in [17]). Through downstream mediators such as MAPK, FAK, PI-3K, Src and PLC-γ, PDGFRs also govern processes such survival, proliferation and angiogenesis (reviewed in [15,18]). The best understood ligands for these receptors include PDGF-A and B which undergo homo- or heterodimerization to become active [16]. In breast cancer, expression of PDGFRs was initially shown to be restricted to the tumor stroma, while ligand was produced by the tumor cells [19,20]. However, recent evidence suggests that expression of both PDGFRα and β, either in the tumor tissue or stroma, is correlated with invasive breast cancers and tumor progression [21-23]. In addition, PDGFRα was shown to be important for maintenance of EMT, evasion of apoptosis, and metastasis in transformed mesenchymal murine mammary cells [22].

The purpose of this study was to examine the role of the PDGF-axis in mammary tumors that survive independent of IGF-IR signaling in MTB-IGFIR mice. Upon IGF-IR suppression, expression of PDGFRα and β were observed to increase, a process which occurred rapidly. In cell lines isolated from IGF-IR-independent recurrent tumors, PDGFRs were shown to facilitate motility and invasion with more modest effects on proliferation and apoptosis.

## Materials and methods

### Animal studies

MTB-IGFIR transgenic mice are described elsewhere [12]. For IGF-IR transgene induction, mice were given a diet containing 2 mg/kg doxycycline (Bio-Serv Inc., Frenchtown, NJ). Tumors were allowed to grow to a maximum length of 17 mm (under the guidelines of the Canadian Council of Animal Care). IGF-IR-independent recurrent tumors were formed after the withdrawal of doxycycline, and are described in [13]. For IGF-IR downregulation studies, mice harboring mammary tumors induced through administration of doxycycline were given a normal diet and tissue was collected 24 and 48 h later. Animals were housed and cared for following guidelines established by the Central Animal Facility at the University of Guelph and the guidelines established by the Canadian Council of Animal Care.

### Cell lines and culture conditions

RM11A cells, a murine mammary tumor cell line derived from an MTB-IGFIR mammary tumor, were grown as described in [24]. Doxycycline (10 μg/mL) was added to the culture media for transgene induction and only omitted from the media for withdrawal studies. RJ345 cells were isolated from primary mammary tumors of MTB-IGFIR mice and cultured in media containing doxycycine as above while RJ348 and RJ423 cells were isolated from IGF-IR-independent spindle recurrent tumors; all of these cell lines were isolated and cultured in media as described in [24]. Tumors formed from all cell lines were generated through injection of approximately 5 × 10^5 ^cells into the 4^th ^mammary gland of syngeneic wild type FVB mice as described in [24]. Tumors were allowed to grow to 17 mm before the animal was euthanized and tissue was collected.

### Western blotting

Western blotting was performed as described in [24]. Briefly, 30 μg of protein lysate was subjected to SDS-PAGE with subsequent transfer onto nitrocellulose membranes. Primary antibodies used included anti-PDGFRα, anti-PDGFRβ, anti-phospho-Akt, anti-Akt, anti-phospho-p42/44, anti-p42/44, anti-Cyclin D1 (all from Cell Signal technology, Danvers, MA, USA), and anti-IGF-IR (R&D Systems, Minneapolis, MN, USA) all used at 1:1000 as well as anti-β-actin (Cell Signaling Technology, Danvers, MA, USA) used at 1:5000. Secondary antibody was anti-rabbit IgG (Cell Signaling Technology, Danvers, MA, USA) or anti-Goat (Santa Cruz, Biotechnologies, Santa Cruz, CA) used at 1:2000. Quantification of protein levels was performed using a FluorChem 9900 imager and AlphaEaseFC software version 3.1.2 (Alpha Innotech, San Leandro, CA, USA).

### Immunohistochemistry

Immunohistochemistry was performed as described in [25]. Sections cut from primary and IGF-IR-devoid recurrent tumors described in [13] were used. Antibodies used were anti-PDGFRα and anti-PDGFRβ; both were used at 1:100 (Cell Signaling Technology, Danvers, MA, USA). Biotinylated anti-rabbit secondary antibody (Sigma-Aldrich, St. Louis, MO) was used for both at a concentration of 1:200. Images were captured using light microscopy.

### Real time PCR

Real time PCR was performed to quantify transcript levels. RNA was isolated using a mirVANA kit (Ambion, Streetsville, ON) in accordance with the manufacturer's instructions (without the miRNA enrichment step). Reverse transcription and real time PCR was performed as described in [13]. Primers specific genes encoding human IGF-IR [13], as well as PDGFRα and PDGFRβ (both obtained from Origene, Rockville, MD) were used. The hypoxanthine-guanine phosphoribosyltransferase (HPRT) gene was used for normalization.

### Transfections and siRNA-mediated protein knockdown

Transient transfections were performed using Lipofectamine (Invitrogen, Burlington, ON) in accordance with the manufacturer's directions. RNA oligonucleotides were transfected at a final concentration of 200 pmol/mL. Briefly, three Stealth siRNA oligonucleotides with binding specificity for murine IGF-IR, PDGFRα or PDGFRβ mRNA (Invitrogen, Burlington, ON) were originally tested for their ability to diminish expression of these gene products through western blotting. An appropriate GC control oligonucleotide (Invitrogen, Burlington, ON) was used as a negative control.

### MTT survival assays

MTT survival assays were performed as described in [24]. Briefly, 500 cells/well were plated in 96-well plates in triplicate. Two days after seeding, cells were transfected with siRNA as indicated above. Three days later, MTT was added to the cells which were subsequently lysed after 1 h. Values are reported relative to the corresponding GC control and represent the average of 5 trials.

### Immunofluorescence

Immunofluorescence was performed as described in [25]. To assess proliferation, cells plated on coverslips were transfected with siRNA oligonucleotides specific for PDGFRα, PDGFRβ or both as described above. Forty-eight hours after siRNA treatement, cells were fixed with 10% formalin for 1 h at room temperature and stained for Ki67. Primary antibody, anti-Ki67 (Abcam, Cambridge, MA), was used at a concentration of 1:200, while antibodies specific for cleaved Caspase-3 and phospho-HistoneH3 (both from Abcam, Cambridge, MA) were both used at 1:100; fluorescent conjugated Alexafluor secondary antibody (Invitrogen, Burlington, ON) was used at a dilution of 1:2000. Images were taken using using an Olympus BX61 fluorescent microscope (Center Valley, PA) with MetaMorph version 7.6.0.0 software (Molecular Devices, Downington, PA) at a magnification of 100×. Positive nuclei from 5-10 fields of view were counted; results represent the average positivity compared to respective controls from three independent trials.

### JC-1 assays

JC-1 assays were employed to test apoptosis after downregulation of PDGFRs. Recurrent cell lines were plated in 12-well plates. Twenty-four hours later cells were transfected with RNAi as outlined above. Forty-eight hours post-transfection, media was removed and fresh media containing JC-1 (Invitrogen, Burlington, ON) at a concentration of 200 μg/mL was added to the cells. Cells were incubated with JC1 for 15 min at 37°C, after which media was removed and wells were washed three times with PBS. Images were captured using an Olympus IX81 microscope (Olympus, Markham, ON) at 200 × magnification using Image Pro Plus V.5.0.2.9 software (MediaCybernetics, Bethesda, MD) with the green fluorescent protein (GFP) channel (485-535 nm).

### Scratch assays

For scratch tests, cells were plated in 12-well plates such that they were 90-100% confluent at the time the scratch was performed. Transient transfection with siRNA (as stated above) was performed 48 h after the cells were seeded. A single scratch was made down the center of the well with a 200 μL pipette tip 4 h post transfection. Subsequently media was removed, cells were washed twice with PBS and media was replaced. Cells were imaged at various timepoints (including immediately after the scratch was produced, 0 h) using an Olympus IX81 microscope (Olympus, Markham, ON) at 100 × magnification and Image Pro Plus V.5.0.2.9 software (MediaCybernetics, Bethesda, MD). This experiment was replicated four times.

### Invasion assays

Recurrent cell lines were transfected with siRNA as described above. Cell invasion was assessed using 8 μm BioCoat™ Matrigel invasion chambers (BD Biosciences, Franklin Lakes, NJ, USA) 24 h after transfection. Chambers were placed in 12-well dishes and the top and bottom compartment were rehydreated with serum free and full serum media (DMEM containing only glutamine, sodium pyruvate, antibiotic/antimycotic and HEPES buffer) respectively for two hours at 37°C. Subsequently, 2.0 × 10^4 ^RJ348 or 1.0 × 10^5 ^RJ423 cells (previously transfected) suspended in either 200 or 500 μL of serum free media were plated in the upper chamber, while 300 μL of fresh full serum media was added to the bottom chamber. Invasion was allowed to proceed for 24 h at 37°C. At this time, 500 μL of 5% gluteraldehyde was added to the bottom chamber and invading cells were fixed for 10 min. Subsequently, wells were washed three times with distilled water and incubated with 0.5% toluidine blue for 20 min. Liquid was then aspirated from both chambers and the lower chamber was washed three times with water. A cotton swab was used to wipe the inner surface of the upper chamber to remove non-invasive cells. Invading cells were visualized and images were captured using an Olympus IX81 microscope (Olympus, Markham, ON) at 20 × magnification and Image Pro Plus V.5.0.2.9 software (MediaCybernetics, Bethesda, MD). Cells were counted from 3-4 fields of view and the average number of invading cells per field of view was expressed relative to respective controls. Results represent the average of three replicates.

### Statistics

Results are presented as the mean ± standard error. Statistical significance was assessed through either a student's *t*-test (for analyzing two means) or analysis of variance (ANOVA) followed by a post hoc Tukey test (for more than two means).

## Results

### PDGFRs are overexpressed in IGF-IR-independent mammary tumors

A previous study from our lab showed that downregulation of the IGF-IR transgene in established mammary tumors in MTB-IGFIR transgenic mice resulted in stable regression of a majority of the tumors, however, a small proportion of the tumors resumed growth and expressed only very low levels of IGF-IR [13]. A DNA microarray was then performed on wild type mammary tissue, mammary tumors induced by high levels of the IGF-IR transgene (also called primary mammary tumors or PMTs) and mammary tumors that resumed growth following IGF-IR transgene downregulation that only expressed low levels of IGF-IR (also called IGF-IR-independent recurrent spindle tumors or RSTs). The microarray data indicated that both PDGFRα and PDGFRβ were upregulated in the IGF-IR-independent, recurrent tumors compared to the IGF-IR-dependent, primary tumors [14]. Based on these findings it was hypothesized that PDGFR overexpression is important for tumor survival following IGF-IR downregulation.

**Figure 1:**
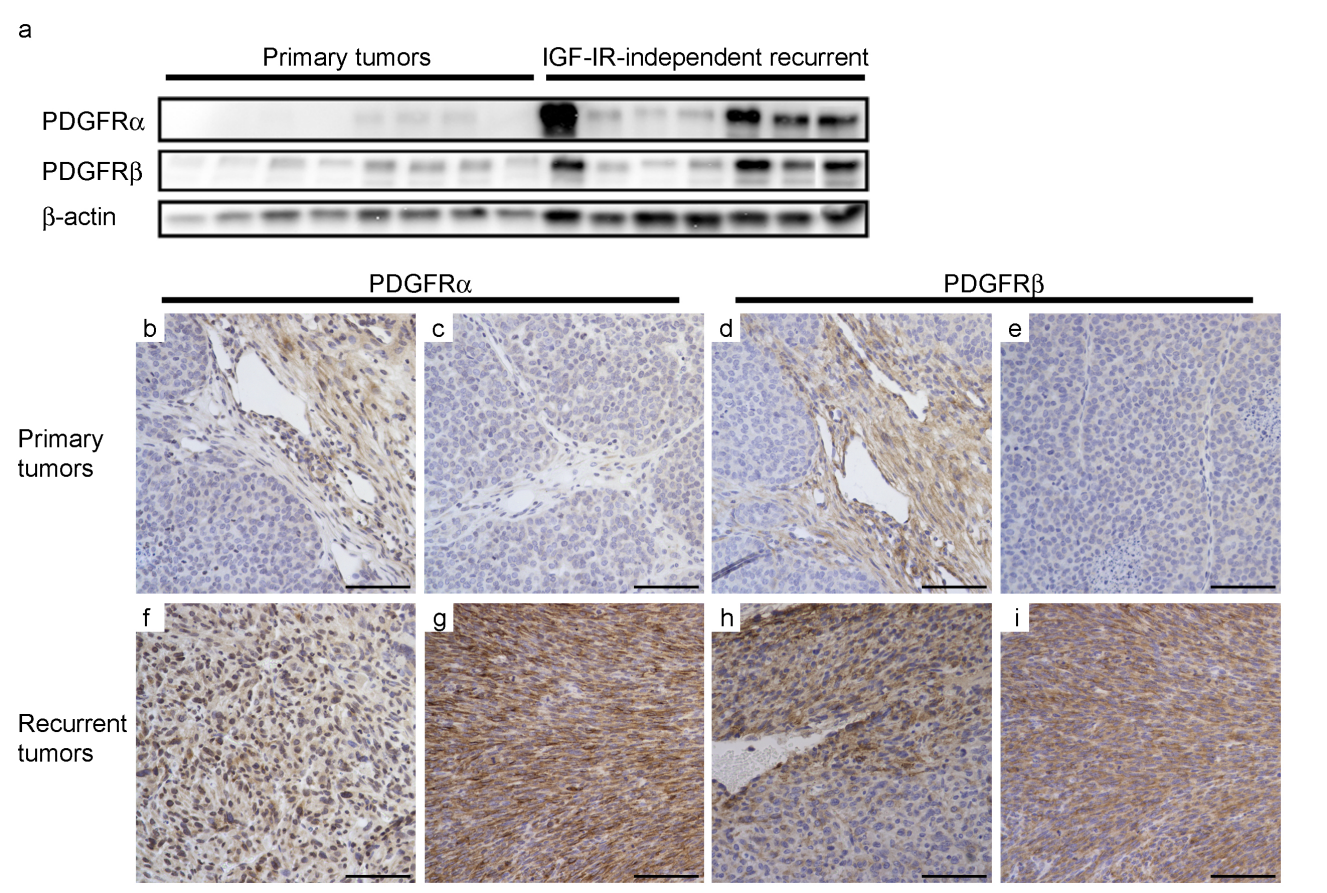
**IGF-IR independence is associated with upregulation of PDGFRs**. Spindle recurrent tumors, formed after doxycycline withdrawal from MTB-IGFIR mice harbouring tumors and subsequent tumor regression, were compared to primary mammary tumors in which the IGF-IR transgene was expressed. Western blotting revealed an upregulation in PDGFRα and PDGFRβ in spindle recurrent tumors (**a**). For the western blots, β-actin served as a loading control. Immunohistochemistry was also used to examine PDGFR expression in two different primary mammary tumors (**b-e**) and in two different IGF-IR independent recurrent tumors (**f-i**). Scale bar = 100 μm.

As a first step, western blotting was performed for PDGFRα and PDGFRβ in primary mammary tumors (IGF-IR-dependent) and IGF-IR-dependent recurrent tumors of MTB-IGFIR transgenic mice. Although there was some inter-tumor variability, the IGF-IR-independent recurrent spindle tumors displayed an overall increase in both PDGFRα and PDGFRβ, with some tumors displaying especially high levels (Figure 1a). Expression of PDGFRs was also assessed using immunohistochemistry. In primary tumors, only sparse expression of both PDGFRα and β was observed (Figure 1b-e) while in IGF-IR-independent recurrent tumors, these receptors were often found to be highly expressed (Figure 1f-i).

### PDGFRs are inversely regulated with IGF-IR expression

**Figure 2:**
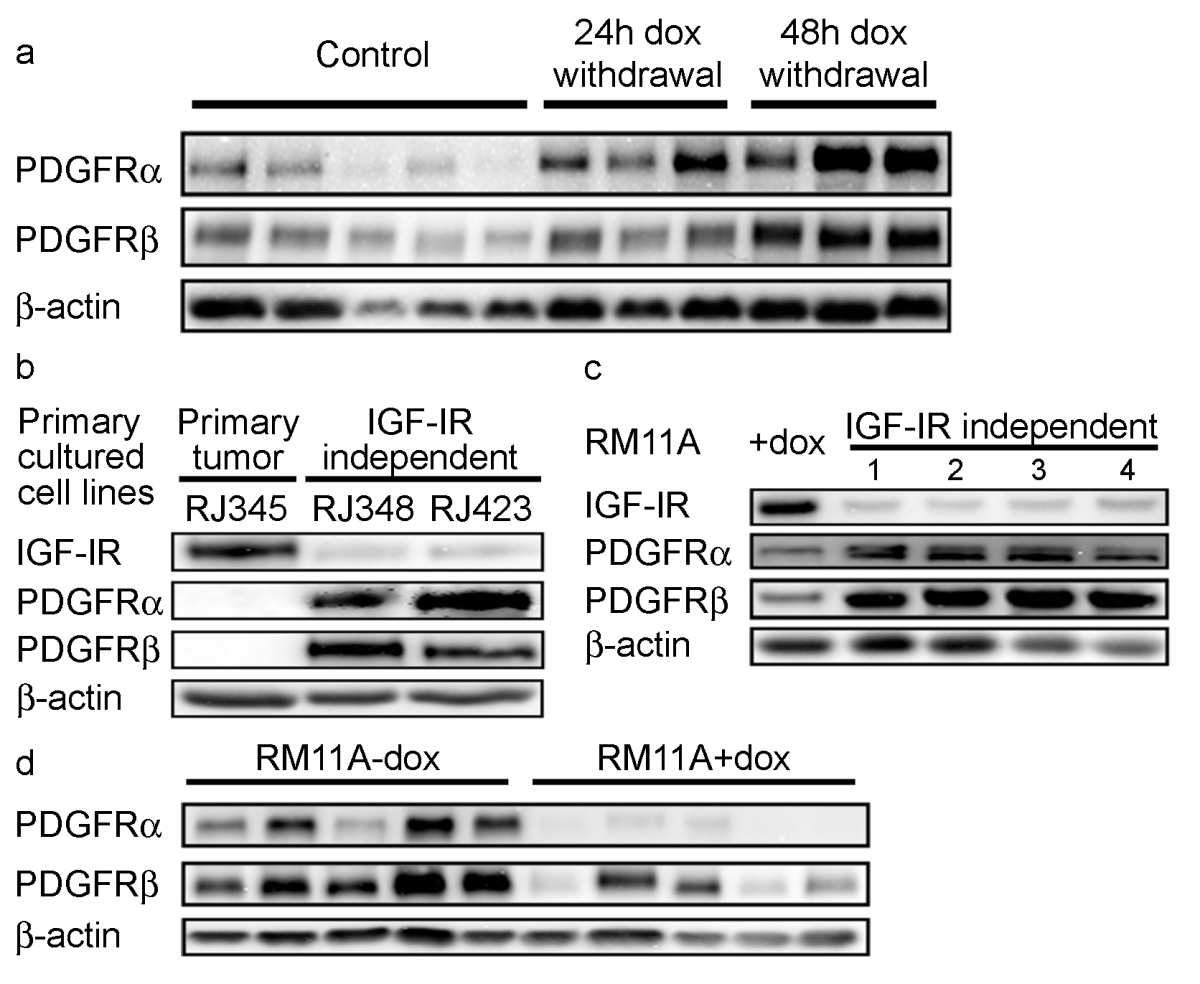
**PDGF receptors are inversely regulated with IGF-IR**. Western blotting was used to examine expression in response to varying IGF-IR levels. Doxycycline was withdrawn from MTB-IGFIR mice with mammary tumors and tissue was collected 24 or 48 h later. Levels of PDGFRs were assessed and compared to that of tumors with IGF-IR induction (**a**). IGF-IR and PDGFR expression in a cell line (RJ345) created from a IGF-IR-dependent primary mammary tumor and two cell lines (RJ348 and RJ348) created from different IGF-IR-independent recurrent spindle tumors (**b**). Four cell clones expressing low levels of IGF-IR were generated through serial passage in the absence of doxycycline. Confirmation of IGF-IR downregulation as well as determination of PDGFRα and PDGFRβ levels was performed (**c**). PDGFR levels in RM11A cells grown *in vivo *with (RM11A + dox) or without (RM11A-dox) doxycycline induction. Tissue was collected when tumors reached 17 mm in diameter (**d**). For all Western blots, β-actin served as a loading control.

To determine if PDGFRs were rapidly upregulated in response to IGF-IR downregulation, MTB-IGFIR transgenic mice with palpable mammary tumors were switched to normal food (not containing doxycycline). IGF-IR levels were previously shown to decrease dramatically at 24 h and 48 h following doxycycline withdrawal [13]. As shown in Figure 2a, both PDGFRα and PDGFRβ were observed to increase at both time points evaluated (24 h and 48 h). Consistent with the western blot data, qRT-PCR analysis revealed that IGF-IR mRNA was significantly decreased in the mammary tumors following doxycycline withdrawal while the levels of PDGFRα and PDGFRβ mRNA were elevated at least 2.9-fold (Table 1).

**Table 1:** Expression of PDGFR transcripts after suppression of IGF-IR levels

RM11A cell line (*in vitro*)	IGF-IR	PDGFRα	PDGFRβ
with doxycycline	1.000 ± 0.000	1.000 ± 0.000	1.000 ± 0.000

without doxycycline	0.042 ± 0.024	1.608 ± 1.087	2.953 ± 0.534

MTB-IGFIR transgenic (*in vivo*)			

Control	1.000 ± 0.223	1.000 ± 0.392	1.000 ± 0.210

24 h doxycycline withdrawal	0.010 ± 0.006	3.113 ± 1.620	3.193 ± 0.948

48 h doxycycline withdrawal	0.004 ± 0.002	7.527 ± 2.475*	2.917 ± 0.818

*Statistically significant compared to control (with doxycycline) *p *< 0.05.

To further validate the induction of PDGFRα and PDGFRβ following IGF-IR transgene downregulation several cell lines were utilized. First, a cell line established from a primary, IGF-IR-dependent mammary tumor (RJ345) and two cell lines from unique IGF-IR-independent recurrent tumors (RJ348 and RJ423) were examined. The RJ345 cells maintained high levels of IGF-IR while the RJ348 and RJ423 cells expressed much lower levels of IGF-IR. Consistent with the *in vivo *findings, tumor cells expressing low levels of IGF-IR (RJ348 and RJ423) expressed high levels of PDGFRα and PDGFRβ compared to the RJ345 cells (Figure 2b).

Next, the RM11A cell line was examined in vitro and *in vivo*. The RM11A cell line was also established from a MTB-IGFIR mammary tumor and this cell line has been more extensively characterized [23]. The RM11A cells express high levels of IGF-IR when cultured in the presence of doxycycline and only express low levels of IGF-IR when cultured in the absence of doxycycline both in vitro and *in vivo *[24]. In addition, the RM11A cells can be injected into the mammary fat pad of syngeneic, FVB mice and these cells form mammary tumors faster in the presence of doxycycline than in the absence of doxycycline [23]. Evaluation of PDGFRα and PDGFRβ in RM11A cells grown in vitro in the presence of doxycycline or in four independent clones grown in the absence of doxycycline for at least four passages revealed that both PDGFRα and PDGFRβ levels increased in the clones expressing low levels of IGF-IR (Figure 2c). A similar increase in PDGFRα and PDGFRβ levels was observed in RM11A cells grown *in vivo *in the absence of doxycycline compared to RM11A cells grown *in vivo *in the presence of doxycycline (Figure 2d).

### Downregulation of PDGFRs in IGF-IR-independent recurrent cell lines results in increased survival

To further evaluate the function of PDGFRα and PDGFRβ in mammary tumor cells that have become independent of IGF-IR signaling, PDGFRα or PDGFRβ were knocked down alone, or in combination, in two IGF-IR-independent mammary tumor cell lines, RJ348 and RJ423. These two cell lines were chosen as they express high levels of PDFGRα and PDGFRβ and low levels of IGF-IR (Figure 2b).

**Figure 3:**
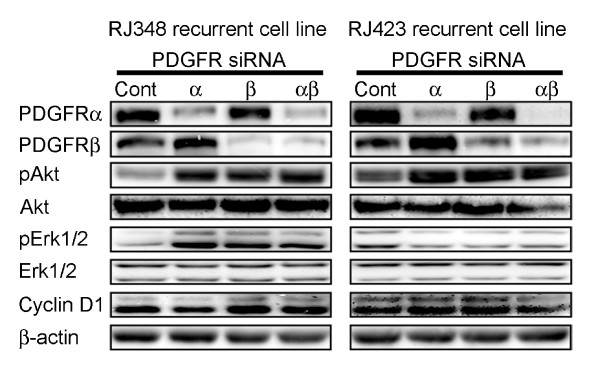
**siRNA mediated downregulation of PDGFRs**. RJ348 and RJ423 recurrent cell lines were plated and 48 h later they were transiently transfected with oligonucleotides specific for either PDGFRα, β or both; an appropriate GC oligonucleotide was used as a control. Seventy-two hours post-transfection, protein was collected and subjected to western blotting probing for both PDGF receptors as well as pAkt, Akt, pErk1/2, Erk1/2 and cyclin D1. β-actin served as a loading control.

Downregulation of PDGFRα or β alone or in combination was consistently achieved in both cell lines and was validated up to 72 h post transfection (Figure 3). Knockdown of either receptor isoform did not result in downregulation of the other. Concomitant administration of siRNA for PDGFRα and PDGFRβ was effective at reducing the expression of both PDFGRs (Figure 3).

Interestingly, with PDGFR knockdown, Akt activation increased in both recurrent cell lines (Figure 3). With pErk1/2, levels increased with PDGFR knockdown in RJ348 cells; however, in RJ423 cells, this protein was observed to have slightly decreased levels. Cyclin D1, an important molecule in our model [12,24], was unchanged after PDGFR downregulation (Figure 3). Three trials were performed to confirm these findings.

**Figure 4:**
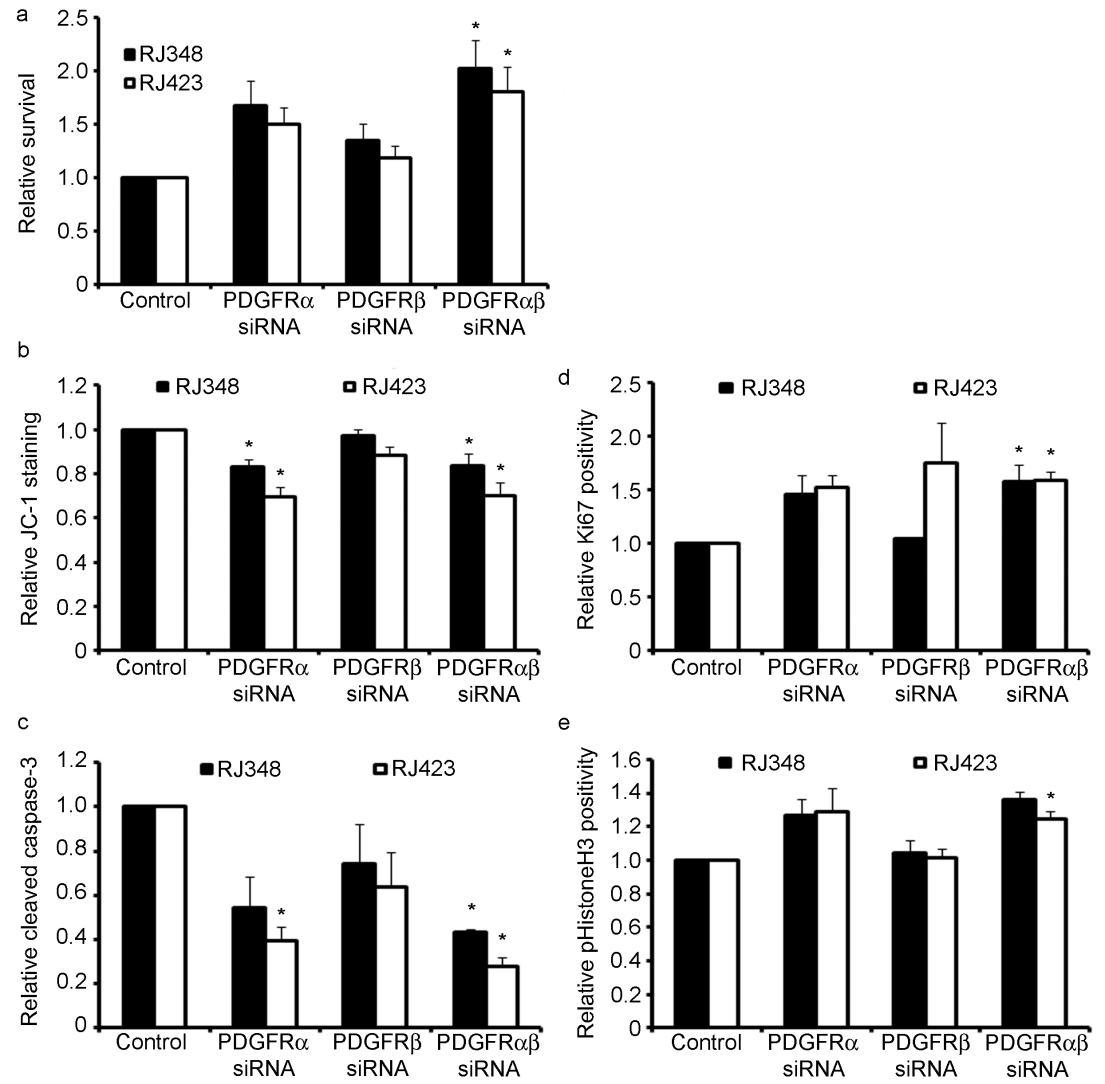
**Downregulation of PDGFRs in IGF-IR-independent recurrent cells increased cell number**. MTT assays were performed to examine cell proliferation/survival after siRNA-mediated knockdown of PDGFRα and β alone or in combination (**a**). Cell number is expressed relative to transfection with the control oligonucleotide and represents the average of five trials. JC1 assays were used to assess apoptosis. Cells were plated in 12-well dishes and transfected with siRNA oligonucleotides specific to PDGFR. JC1 was added after 48 h and fluorescent green cells were quantified. Results represent the average of 3-4 trials relative to controls (**b**). Apoptosis was also evaluated by immunofluorescence staining for cleaved Caspase-3 (**c**). Proliferation was quantified using immunofluorescence staining for Ki67 (d) and phosphorylated HistoneH3 (**e**). For all three above assays, cells were plated in 6-well dishes on coverslips and transfected as above. Fixing and staining was performed 48 h post transfection. Images were collected at 100 × magnification and positive nuclei were counted. Results represent the average percent positivity relative to controls for three individual trials (**d, e, f**). * = *p *< 0.05 (relative to respective control).

It was expected that given the dramatic increase in PDGFR expression in recurrent tumors and cells, this pathway would be important for cell proliferation and survival. To test this hypothesis, MTT assays were performed. Contrary to expectations, cell number increased by approximately 1.7 and 1.5-fold with PDGFRα knockdown in RJ348 and RJ423 cells, respectively. A more modest 1.4 and 1.2-fold increase in cell number was observed with downregulation of PDGFRβ, while dual knockdown increased cell number by 2.0 and 1.8-fold in RJ348 and RJ423 cells, respectively (Figure 4a). To determine if this effect was due to changes in proliferation or apoptosis, subsequent assays were performed.

Apoptosis was addressed by using both JC-1 assays and staining for cleaved caspase-3 after knocking down PDGFRs. JC1 assays are based on the detection of loss of mitochondrial membrane potential, a hallmark of apoptosis, using a lipophilic cationic dye 5,5', 6,6'-tetrachloro-1,1',3,3' -tetraethylbenzimidazolcarbocyanide dye (JC-1); this dye naturally exhibits a green fluorescence during low membrane potential, but changes to red fluorescence as membrane potential increases. Thus cells staining intensely green are counted as apoptotic [26]. As seen in Figure 4b and 4c, knockdown of PDGFRα resulted in a substantial decrease in apoptosis in both cell lines, while PDGFRβ downregulation resulted in a more modest insignificant decrease in apoptosis. Little further decrease in apoptosis, compared to PDGFRα knockdown, was observed when both isoforms were targeted as assessed through both aforementioned assays (Figure 4b, c).

Proliferation after PDGFR downregulation was quantified through staining for Ki67, a well-known marker of proliferation, and phosphorylated HistoneH3. As seen in Figure 4d and 4e, PDGFRα as well as dual PDGFR knockdown resulted in an almost identical increase in relative Ki67 and pHistoneH3 positivity of approximately 1.6-fold and 1.3-fold respectively in both cell lines. In RJ348 cells, PDGFRβ had no effect on Ki67 positivity. In RJ423 cells, knockdown of this receptor isoform had variable results with an average overall increase in Ki67 staining of approximately 1.8-fold (Figure 4d). PDGFRβ knockdown had only a minimal effect on phospho-HistoneH3 staining in both cell lines (Figure 4e). Only downregulation of both PDGFR isoforms resulted in a significant increase in proliferation (Figure 4d, e).

### PDGFR knockdown in recurrent cell lines impairs migration and invasion

**Figure 5:**
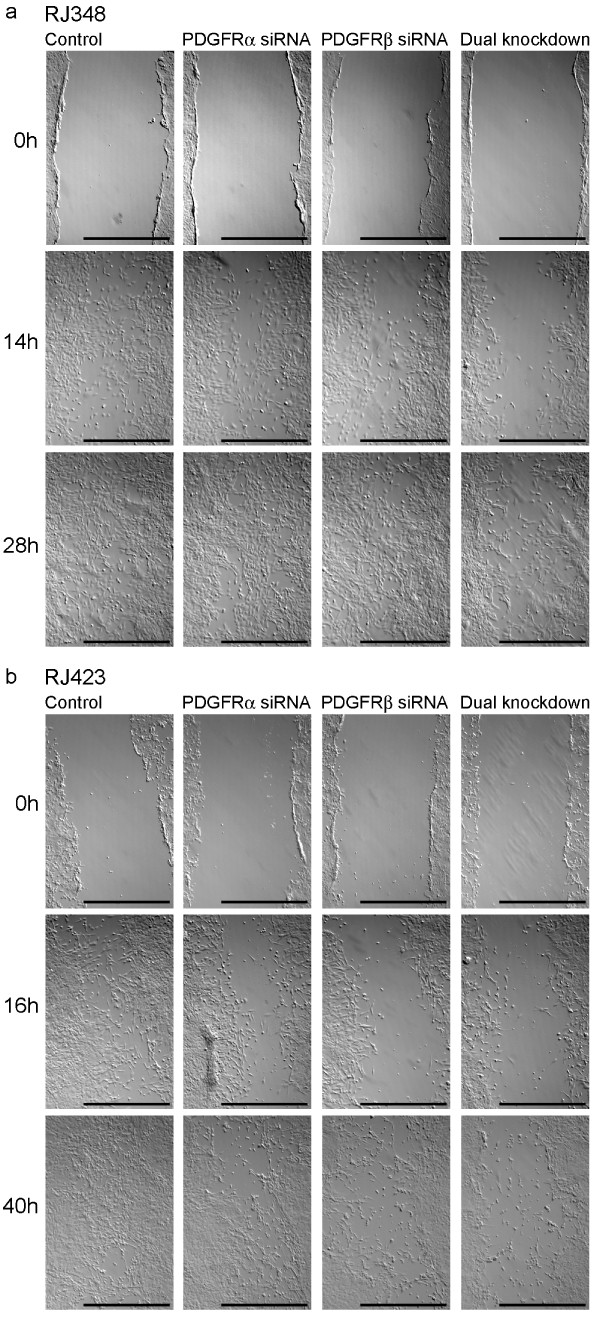
**PDGFR knockdown inhibited cell migration in IGF-IR-independent recurrent cells**. PDGFRα and β were knocked down transiently using siRNA alone or in combination 48 h after plating either RJ348 or RJ423 cells (depicted in a and b respectively). Four hours later a pipette tip was used to scratch a wound down the middle of the cultured cells (time 0 h, depicted above). Images were captured at 40 × magnification using an inverted bright field microscope at varying time points after the scratch was made. Representative results are shown; the assay was repeated four times. Scale bars = 1 mm.

To assess the importance of PDGFRs for cell migration in these recurrent cell lines, scratch wound assays were employed after downregulation of PDGFRs. Images were captured at various timepoints after the scratch wound was made and images shown are representative of four trials (Figure 5). As shown, in the RJ348 cell line, control cells had penetrated the wound by 14 h and wounds were observed to be virtually filled by 28 h. With knockdown of either PDGFRα or β, a slight impairment was observed after 14 h; however, after 28 h no differences were seen compared to control cells. When both receptor isoforms were downregulated, cells did not begin to fill the wound until after 14 h and even after 28 h, many large gaps devoid of cells were observed (Figure 5a). For RJ423 cells a similar trend was observed; a dramatic reduction in migration was observed after knocking down individual PDGFR isoforms or both isoforms at the first timepoint, 16 h. At the second timepoint, 40 h, control cells had almost completely filled the wound; with knockdown of either PDGFRα or β, relatively large gaps were still observed and with dual knockdown, very little progress had been made in filling the wound (Figure 5b). With knockdown of either PDGFR isoform, it took approximately 45 h for the wound to be completely filled, while with dual knockdown, wounds finally were filled by 72 h (data not shown).

**Figure 6:**
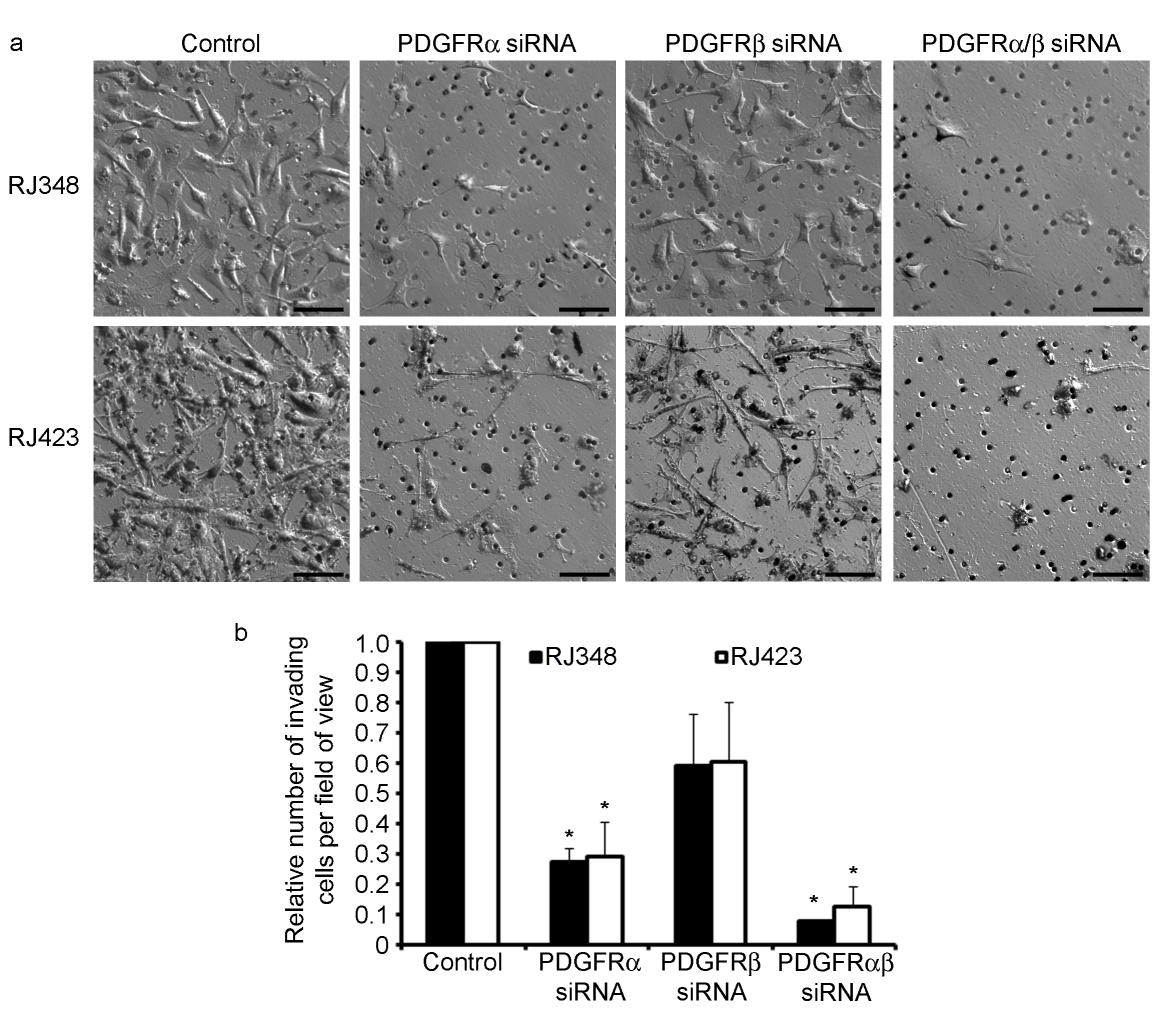
**Knockdown of PDGFRs inhibited invasion in recurrent cell lines**. Matrigel coated chambers were used to assess cell invasion after PDGFR siRNA. Cells were transfected with siRNA and 24 h later, cells were lifted, resuspended in serum free media and added to the upper chamber of the matrigel insert; full serum media was added to the bottom well. After 24 h, cells passing through the matrigel coated membrane were fixed and stained with toluidine blue. Representative images for each condition are shown (**a**). Scale bar = 50 μm. The average number of invading cells per field of view was calculated and related back to respective controls (**b**). Results represent the average of 3 replicates. * = *p *< 0.05.

A system utilizing matrigel coated membranes was employed to assess the role of PDGFRs in extracellular matrix (ECM) invasion in these recurrent cells (Figure 6a). Downregulation of PDGFRα in both cell lines resulted in a statistically significant decrease in invasion of approximately 70%, while PDGFRβ knockdown inhibited invasion by approximately 40% (not determined to be statistically significant) (Figure 6b). Dual knockdown of both PDGFR isoforms resulted in an even greater increase in invasion inhibition compared to downregulation of either isoform alone (92% and 88% inhibition for RJ348 and RJ423 respectively).

## Discussion

While IGF-IR-targeting drugs hold promise for the treatment of breast cancer, based on what is known of other such targeted therapeutics, resistance is sure to be a major roadblock for the treatment of certain patients. Our work on the MTB-IGFIR transgenic mice supports the hypothesis that some tumors, although initially dependent on IGF-IR signaling for growth, can become independent of IGF-IR signaling. We found that downregulation of the IGF-IR transgene (through removal of doxycycline) in established mammary tumors led to the regression of most of the mammary tumors. However approximately 11% of the tumors resumed growth following IGF-IR transgene downregulation and these tumors no longer expressed high levels of IGF-IR [13]. Analysis at both the mRNA and protein level revealed that PDGFRα and PDGFRβ were consistently upregulated in tumor cells and tumor tissue following loss of IGF-IR expression. Therefore, one change that occurs during IGF-IR independent tumor growth is the upregulation of PDGFR signaling.

Although this reciprocal expression pattern has not been described previously in breast cancer, it has been observed in a rhabdomyosarcoma cell line selected for resistance to BMS-754807 (an IGF-IR small molecule inhibitor) [27]. In this study, administration of increasing concentrations of BMS-75807 to Rh41 cells for approximately 5 months resulted in the generation of a clone resistant to BMS-754807. The BMS-754907-resistant clone expressed lower levels of IGF-IR and elevated levels of PDGFRα compared to the parental cells. In addition, the investigators found that the combination of an anti-IGF-IR agent with an anti-PDGFRα agent resulted in synergistic cell kill [27]. A reciprocal relationship between IGF-IR and PDGFRα/c-kit expression has also been observed in gastrointestinal stromal tumors [28]. Therefore, it appears that PDGFR signaling is enhanced following the loss of IGF-IR signaling and vice versa, an important concept to considering when designing therapeutic strategies targeting either of these receptors.

To determine the functional consequence of elevated PDGFRα and PDGFR β, cell survival, proliferation, apoptosis and migration/invasion were evaluated following RNAi-mediated knockdown of PDGFRα and PDGFR β alone or concomitant knockdown of both receptors. The most dramatic effect was the decrease in migration and invasion following concomitant knockdown of both PDGFRs. In other systems, PDGFRβ has previously been shown to promote cell migration, while PDGFRα has been shown to inhibit this process [29-31]. However, more recently, it was shown that both receptors could promote migration in fibroblasts with additive effects when both were present [18]. Similar to these results, the greatest inhibitory effect on migration was observed in this study when both isoforms were knocked down compared to either one individually. Analogous to our findings, in human breast cancer cell lines, PDGF ligands have also previously been shown to mediate invasion or upregulate markers of invasion [32,33]. These processes are important for metastasis and it will be interesting to determine if the PDGF-axis is involved in metastasis in our model, especially during IGF-IR-independent recurrence. Previously, in a model of ras-transformed mammary epithelial cells undergoing EMT, PDGFR inhibition was shown to impede metastasis [22].

Somewhat surprisingly, downregulation of both PDGFRs resulted in a modest increase in proliferation and a decrease in apoptosis. These results are not in accordance with conventional models of PDGFR signaling. Typically this pathway is involved in survival and proliferation for mesenchymal cells including a number of cancers of mesenchymal origin such as dermatofibrosarcoma protuberans, gastrointestinal stromal tumors, chronic myeloid leukemias and gliomas [15,34]. However, the mitogenic and transforming effect of PDGFRs in other tumor types of epithelial origin, including breast cancer, remains largely unknown. While targeting the PDGFR usually results in inhibition of cell survival [35], similar results to ours have been reported in other models; PDGFRα overexpression was shown to inhibit melanoma tumor cell growth *in vitro *and *in vivo *[36], while overexpression of PDGF-BB in human pancreatic and colorectal cells resulted in a reduction in tumor growth [37].

Interestingly, the levels of phosphorylated Akt were elevated in both cell lines following downregulation of PDGFRs. This observation is in contrast to published reports indicating that PDGFRs signal via Akt [38], but would explain why an increase in proliferation and a decrease in apoptosis was observed following PDGFR downregulation. Phosphorylated Akt initiates signals that promote cell proliferation and inhibits apoptosis [39-41]. Therefore, activation of a signaling pathway other than the PDGFRs must compensate for the loss of IGF-IR signaling and maintain high levels of Akt phosphorylation in our model. One logical receptor family would be the EGFR/ErbB family as these receptors have been shown to mediate resistance to IGF-IR targeting agents in some models [42-44]. However, western blotting of IGF-IR-dependent and IGF-IR-independent mammary tumors did not show any consistent differences in the levels of EGFR, ErbB2 or ErbB3 or the phosphorylated forms of these receptors (data not shown).

Recently we have shown that mammary tumors that become independent of IGF-IR signaling in MTB-IGFIR transgenic mice and the RJ348 cell line have characteristics of claudin-low breast tumors [14,45]. The claudin-low breast cancer subtype was identified in 2007 and is characterized by low levels of claudins 3, 4 and 7 as well as other tight junction proteins [46,47]. Claudin-low tumors also express high levels of markers associated with epithelial-to-mesenchymal transition (EMT) such as Twist1/2, Zeb1/2, Slug and Snail while expressing little or no markers of luminal differentiation [46]. The prevalence of claudin-low tumors is reported to be 7-14% and claudin-low tumors have a prognosis similar to luminal B, HER2 enriched and basal-like breast cancers [47]. Claudin-low subtypes have been reported to most closely resemble mammary epithelial stem cells [47]. Therefore, it is possible that claudin-low tumors present with elevated levels of PDGFRα and PDGFRβ, a characteristic that may improve the treatment of this class of tumor.

In conclusion, loss of IGF-IR signaling is associated with an increase in PDGFRα and PDGFRβ expression. This increase in PDGFRs appears to enhance migration and invasion of mammary tumor cells. This phenomenon could have important clinical implications in that treatment with anti-IGFIR agents may result in an increase in PDGFR expression and enhanced metastatic capacity of breast cancer cells. Therefore, it may be important to combine anti-IGF-IR strategies with agents inhibiting PDGFR signaling to effectively eradicate tumor cells and to prevent metastasis of any tumor cells that become resistant to the anti-IGFIR therapy. Alternatively, elevation of PDGFRs may be a characteristic of claudin-low tumors and this feature may be exploited to improve the treatment of this breast cancer subtype.

## Competing interests

The authors declare that they have no competing interests.

## Authors' contributions

CC participated in design and coordination of the study as well as all of the experiments described in this study and drafting of the manuscript. RM coordinated the study and contributed to drafting of the manuscript. All authors have read and approved the final manuscript.

## Pre-publication history

The pre-publication history for this paper can be accessed here:

http://www.biomedcentral.com/1471-2407/11/480/prepub
